# Supplementary material for: Isoflavones Mediate Dendritogenesis Mainly through Estrogen Receptor α
Source: Int J Mol Sci. 2023 May 19;24(10):9011. doi: 10.3390/ijms24109011 (PMC10218940; doi:10.3390/ijms24109011)
Supplement: Supplementary file 1 [file ijms-24-09011-s001.zip › S1.pdf]

A. ER and GPER1 expression in Primary cerebellar culture after ER or GPER1 knockdown

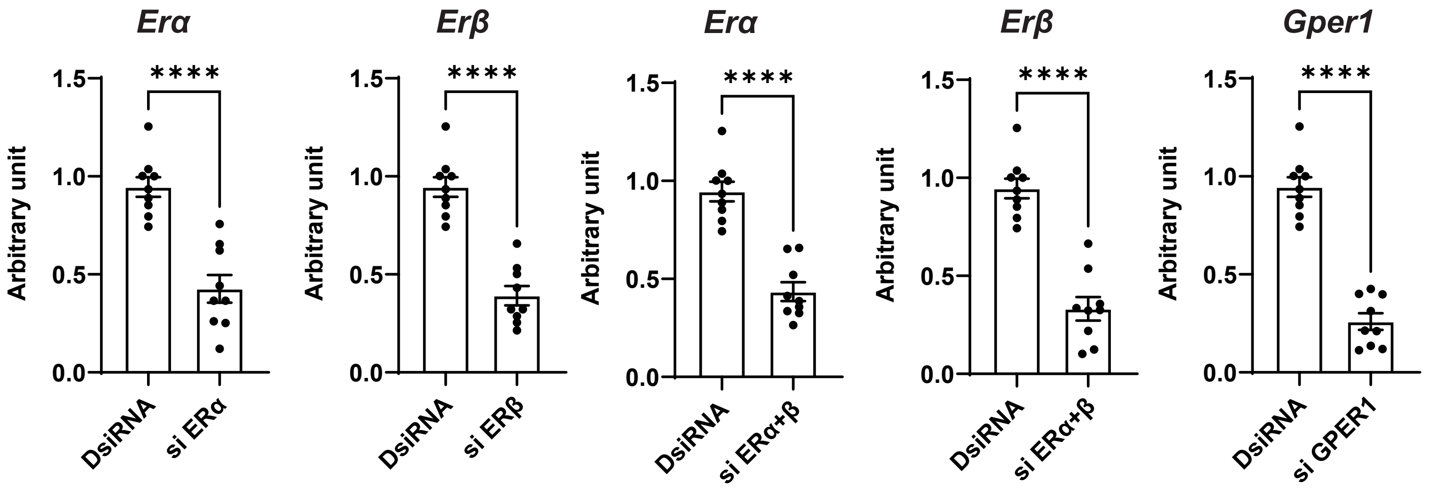

B. ER and GPER1 expression in Neuro-2A cells after ER or GPER1 knockdown

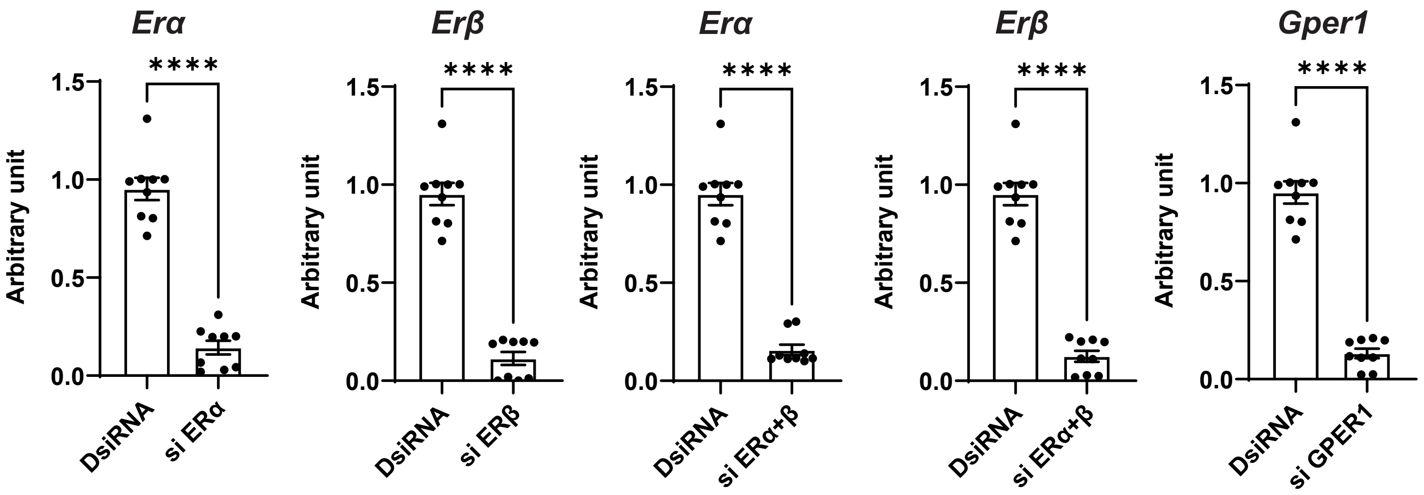

Supplementary Figure S1.
