# Supplementary material for: Isoflavones Mediate Dendritogenesis Mainly through Estrogen Receptor α
Source: Int J Mol Sci. 2023 May 19;24(10):9011. doi: 10.3390/ijms24109011 (PMC10218940; doi:10.3390/ijms24109011)
Supplement: Supplementary file 1 [file ijms-24-09011-s001.zip › Supplementary Table.pdf]

Supplementary Table.

Table S1. siRNA target sequences.

Gene	Sense	Antisense
DsiRNA Controls	CGUUAAUCGCGUAUAAUACGCGUAT	AUACGCGUAUUAUACGCGAUUAACGAC
ERα (Mouse)	ACAGUUGACUUCAUAAGC	UUCAGUAGCUUAUGAAGU
ERβ (Mouse)	CUAGGCAAGAACAUUACC	AUCAGUUGGUAAUGUUCU
GPER (Mouse)	GCAUCCUGGAUGGAAUUC	UUCACUUCAAUUCCAUCC

Table S2. Primer sequences.

Gene	Sense	Antisense
Erα (Mouse)	TGCGCAAGTGTTACGAAGTG	TCTGACGCTTGTGCTTCAAC
Erβ (Mouse)	AGGAATGGTCAAGTGTGGATCC	TGGCTTTGTTCAGGCAATGC
Gper (Mouse)	TTCAACCTGGACGAGCAGTAC	AGACGCTGCTGTACATGTTG
Bdnf (Mouse)	ATCCAAAGGCCAACTGAAGC	ATTGGGTAGTTCGGCATTGC
Camk2b (Mouse)	TGCAAGGAGGAAGCTCAAGG	CTGTTTGTCTGGGGCTTGAC
Rbfox3 (Mouse)	ACAGACAGACAACCAGCAAC	CGAATTGCCCGAACATTTGC
Tubb3 (Mouse)	AGCTGTTCAAACGCATCTCG	GACACCAGGTCATTCATGTTGC
Syn1 (Mouse)	TGTGCGTGTCCAGAAGATTG	ACATGGCAATCTGCTCAAGC
Dlg4 (Mouse)	GGTCAACGACAGCATCCTG	ATGACGTAGAGGCGAACGATG
Syp (Mouse)	TTTGCCATCTTCGCCTTTGC	GGGTGCATCAAAGTACACTTGG
Map2 (Mouse)	ATGAAGGAAAGGCACCACAC	TGGAAATCCATTGGCGTTGC
Gapdh (Mouse)	TGCGACTTCAACAGCAACTC	ATGTAGGCCATGAGGTCCAC
